# Complexity of the Tensegrity Structure for Dynamic Energy and Force Distribution of Cytoskeleton during Cell Spreading

**DOI:** 10.1371/journal.pone.0014392

**Published:** 2010-12-21

**Authors:** Ting-Jung Chen, Chia-Ching Wu, Ming-Jer Tang, Jong-Shin Huang, Fong-Chin Su

**Affiliations:** 1 Institute of Biomedical Engineering, National Cheng Kung University, Tainan, Taiwan; 2 Department of Cell Biology and Anatomy, National Cheng Kung University, Tainan, Taiwan; 3 Department of Physiology, National Cheng Kung University, Tainan, Taiwan; 4 Department of Civil Engineering, National Cheng Kung University, Tainan, Taiwan; University of Oxford, United Kingdom

## Abstract

Cytoskeleton plays important roles in intracellular force equilibrium and extracellular force transmission from/to attaching substrate through focal adhesions (FAs). Numerical simulations of intracellular force distribution to describe dynamic cell behaviors are still limited. The tensegrity structure comprises tension-supporting cables and compression-supporting struts that represent the actin filament and microtubule respectively, and has many features consistent with living cells. To simulate the dynamics of intracellular force distribution and total stored energy during cell spreading, the present study employed different complexities of the tensegrity structures by using octahedron tensegrity (OT) and cuboctahedron tensegrity (COT). The spreading was simulated by assigning specific connection nodes for radial displacement and attachment to substrate to form FAs. The traction force on each FA was estimated by summarizing the force carried in sounding cytoskeletal elements. The OT structure consisted of 24 cables and 6 struts and had limitations soon after the beginning of spreading by declining energy stored in struts indicating the abolishment of compression in microtubules. The COT structure, double the amount of cables and struts than the OT structure, provided sufficient spreading area and expressed similar features with documented cell behaviors. The traction force pointed inward on peripheral FAs in the spread out COT structure. The complex structure in COT provided further investigation of various FA number during different spreading stages. Before the middle phase of spreading (half of maximum spreading area), cell attachment with 8 FAs obtained minimized cytoskeletal energy. The maximum number of 12 FAs in the COT structure was required to achieve further spreading. The stored energy in actin filaments increased as cells spread out, while the energy stored in microtubules increased at initial spreading, peaked in middle phase, and then declined as cells reached maximum spreading. The dynamic flows of energy in struts imply that microtubules contribute to structure stabilization.

## Introduction

The biological functions of cells, such as differentiation, growth, metastasis, and apoptosis are associated with cell shape, which is related to the mechanical forces in the cytoskeleton [1], [2], [3], [4]. Cytoskeleton, the major mechanical component of cells, supports the cell architecture and dominates cell motility by performing contractility. The cytoskeleton also transmits mechanical stimulation for intracellular signal transduction [5], [6], [7]. Several cytoskeleton models investigated the mechanical properties of cells using computational stimulations [1], [4], [8], [9], [10], [11], [12]. The prestressed cable net [8], [10] and semi-flexible chain net [11] are used to form actin cytoskeleton model for prediction of cell stiffness under mechanical perturbations in two-dimensions. Although the prestressed cable net [4] and open-cell foam model [12] constructed three-dimensional (3-D) cytoskeletal models, the simulations only considered tensile elements (actin filaments). The tensegrity [1], [7] and granular model [9] comprise tensile elements and compressive elements (microtubules) that providing cell stability and intracellular force equilibrium [13], [14].

Cytoskeleton models mostly concentrated on evaluating cell elasticity against cell deformation or material properties of cytoskeletal constituents [1], [8], [11]. Although rheological responses of cells by changing prestress were modeled previously [15], [16], [17], the dynamic simulation of cell behavior still receives little attention. Tensegrity is a structure composed of continuous cables and discrete struts. Cables represent actin filaments and bear tensile forces, whereas struts represent microtubules and only stand compressive forces. Different complexities of tensegrity structures are constructed by different layers of cable-strut net [18]. Previous studies commonly employed the simple octahedron tensegrity (OT) structure, comprising of 24 cables and 6 struts with 12 jointed nodes [1], [3], [15], [16], [19], [20]. The cuboctahedron tensegrity (COT), a more complicated structure, is made of 48 cables, 12 struts, and 24 jointed nodes [21]. To describe both tensile and compressive properties of cells, the present study applied the tensegrity structure to develop numerical models.

A successful simulation requires a reliable model to describe cell behavior and predict intracellular conditions. This study aimed to develop a 3-D cytoskeleton model with a spreading morphology to describe cell behavior. Two tensegrity structures, OT and COT, were adopted to reflect the different complexity of cytoskeleton models. Different degrees of cell spreading were applied to test the sufficiency of structure complexity by considering the equilibrium and the stability in tensile and compressive elements. The strain energy of cytoskeleton was studied for choosing the optimized simulated structure by minimizing energy consumption. The distribution of traction forces on focal adhesions (FAs) was also demonstrated for simulating the living cell features. The COT structure provided superior results for numerical simulations. The findings of this study pertain the structure arrangement to the observations in cytoskeleton and interpret the spreading mechanism in living cells, thereby ascertaining the reasonableness of using COT structure as the spreading cytoskeleton models.

## Methods

### Materials

The simulation and analyses of cell spreading were performed using the commercial finite element package ABAQUS (standard version 6.6, SIMULIA). The simulation was conducted using a personalized computer (Acer Inc., Taiwan) with an Intel processor (2.66GHz) and 3.25GB of RAM. Simulated data were stored on a 500GB hard drive (Western Digital).

### Tensegrity Properties for Cytoskeleton

Cables and struts in the tensegrity structure represented actin filaments and microtubules, respectively. Tensegrity, a prestressed and self-equilibrated structure, consisted of pre-tensed actin filaments and pre-compressed microtubules equilibrating each other without external support in the un-deformed states [1], [3], [22]. The nodes were pinned and denoted as candidates for FAs. Both the OT and COT structures were sphere-like structures and represented a hollow structure in their un-deformed states. The easily folded structure could deform to describe the change of cell shapes under different conditions.

**Figure 1:**
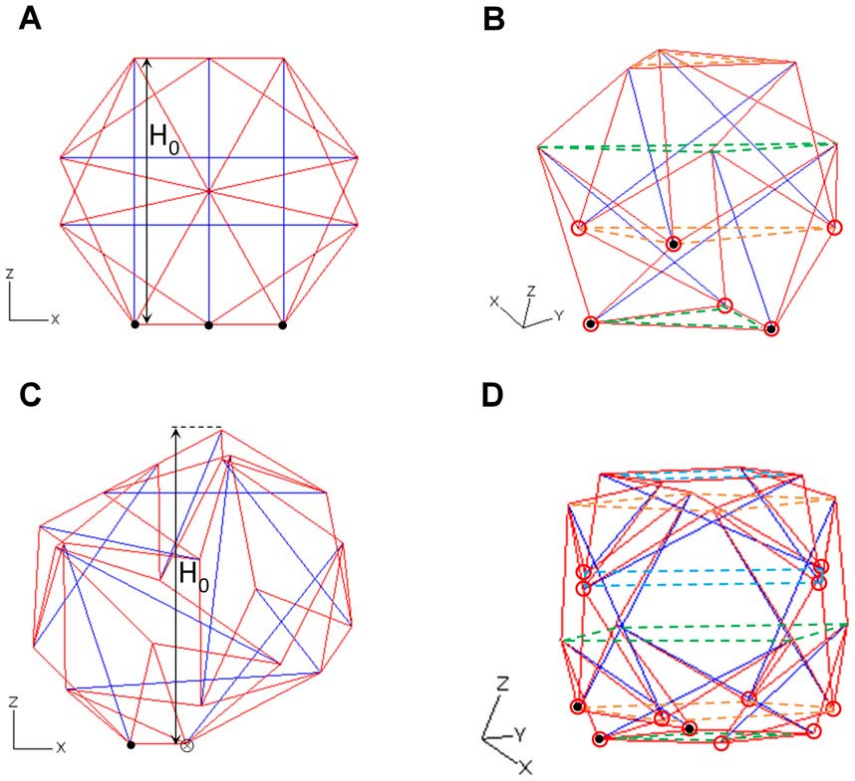
Two spherical tensegrity structures with different complexities. The octahedron tensegrity (OT) is composed of 24 cables (red) and 6 struts (blue) (A). An initial height 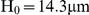 is measured on the X-Z plane. Two pairs of triangular planes (green and orange dash lines) separated the OT structure into two overlapping layers (B). 48 cables (red) and 12 struts (blue) formed the cuboctahedron tensegrity (COT) structure with an initial cell height 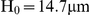 (C). The COT structure is a three-layer structure separated by three pairs of square planes (green, orange and blue dash lines) (D). The candidates for attaching nodes during spreading are marked as red circles in both the OT (B) and COT (D) structures. The initial boundary condition for each structure has three nodes (solid black circles) attached on the rigid floor (x-y plane) (A and C). ⊗ means one node overlaps another in the view direction.

To build the OT structure, the relative positions of 6 struts with a length of 16µm were first defined using ABAQUS as described previously [18]. Each pair of parallel struts formed a plane, and the 6 struts established three orthogonal planes (blue element, Fig. 1A). Then, the ends of neighboring struts were connected with a length of 9.8µm to establish 24 cables (red element, Fig. 1A). The OT structure used 12 nodes and was employed as a cytoskeleton model based on aforementioned cell-like features (Figs. 1A–B) [12].

For the COT structure, 12 struts with a length of 12µm were drawn using ABAQUS and rearranged to their relative positions [22], which induced four planes intercrossing at the structure center (blue element, Fig. 1C). Then, 24 nodes connected cables at both ends of the struts. A total of 48 cables were drawn by connecting the ends of neighboring strut with two different lengths of 7.14 and 6.24µm (red element, Fig. 1C). Among the 48 cables, the longer cables (7.14µm) composed the square patterns (the green dash square at the bottom), whereas the shorter cables (6.24µm) formed the triangle patterns (Fig. 1D). After construction, tensegrity structures were rotated and translated to determine initial boundary conditions.

### Material Properties of Elements

Cables and struts were assumed to behave linear-elastically to clarify the contributions of actin filaments and microtubules with respect to their mechanical properties during cell spreading. The Young's modulus of cables and struts was 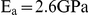 and 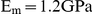, respectively, in accordance to the experimental measurements [9], [19], [23]. The tensile force carried in a cable, F, with a current length 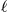 is:
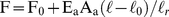
(1)where, 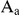 is the cross-section area of cables and was 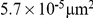 (solid cylinder with a radius of 4.25 nm) [24]. 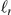 denotes the resting length of cables, whereas 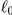 denotes the initial length of cables in a tensegrity structure without deformation.

The compressive force carried in a strut, P, with a current length L is:
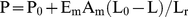
(2)where, 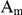 is the cross-section area of the struts and was 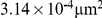 (hollow cylinder with an outer diameter of 25 nm and an inner diameter of 15 nm). 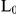 and 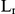 denote the initial and resting length of the struts, respectively. The lengths of cables and struts have a relationship of 
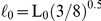
 in the OT structure (Fig. 1B). The geometrical relationships in the COT structure are 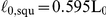 and 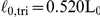. The subscript “squ” stands for cables comprising square patterns and “tri” stands for cables comprising triangle patterns (Fig. 1D).

The dimensions of constitutive elements in the OT structure were 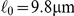 and 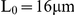, which led to an initial structure height of 14.3µm (Fig. 1A). The COT structure had a height of 14.8µm with 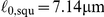, 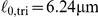, and 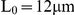 (Fig. 1C). 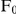 and 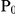 in Equations (1) and (2) describe the initial pre-tension of cables and pre-compression of struts in an undeformed tensegrity. In general, 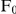 is equal to the force produced by a single actomyosin unit measured roughly from 0.2 to 6pN [25]. 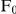 had an average value of 1.6pN for the OT structure in the current study. 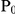 then became 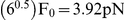 when achieving an initial self-equilibrated status [18], [26]. In the COT structure, the initial pre-tension of cables composing triangle patterns, 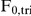, was 1.6pN. For the self-equilibrated status, the pre-tension of cables composing square patterns, 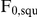, was 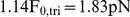. The pre-compression of struts was 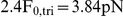 [22]. The mechanical settings of the elements in both the OT and COT tensegrity structures are summarized in Table 1.

**Table 1:** Mechanical settings for cytoskeletal elements in octahedron and cuboctahedron structures.

Category	Subcategory	Octahedron	Cuboctahedron
**Amount**	Nodes	24	48
	Cables	12	24
	Struts	6	12
**Initial length (µm)**	Cables	9.8	6.24 (with triangle patterns)7.14 (with square patterns)
	Struts	16	12
**Radius of element cross-section (nm)**	Cables	4.25	4.25
	Struts	Inner:7.5Outer:12.5	Inner:7.5Outer:12.5
**Young's modules (GPa)**	Cables	2.6	2.6
	Struts	1.2	1.2
**Initial pre-force of elements (pN)**	Cables (tension)	1.60	1.60 (with triangle patterns)1.83 (with square patterns)
	Struts (compression)	3.92	3.84
**Initial height (µm)**		14.3	14.8

### Spreading Principles in Dynamic Simulation

During the spreading simulation, the cables and struts were depicted as truss elements that only supported axial force and deformation. The initial boundary condition for the OT and COT structure had two cables lying on the x-y plane and three nodes pinned to the x-y plane (solid black circles, Figs. 1A and 1C). The substrate (x-y plane, where the cytoskeleton structures attached) was assumed to be a rigid floor to ignore mechanical interactions between cytoskeleton forces and substratum rigidity. The nodes connected with cables and struts were pinned as free movable joints. Half of the connected joints were candidates for attaching nodes and formed FAs in a spread out structure. When the structure reached the maximum numbers of FAs, one end of every strut was attached to the x-y plane. The FAs were allowed to move to the new location on the x-y plane and other nodes were free of constraints.

The nodes located at the lower end of each strut were candidates for FAs (hollow red circles, Figs. 1B, 1D); therefore, the maximum number of attached nodes was six in OT structure and twelve in COT structure. Three candidates were chosen to pin on the x-y plane using ABAQUS (Figs. 1A, 1C). In the first step of spreading OT structure, a candidate closest to the attachment plane was moved to form an FA on the x-y plane. To ascertain minimum force during FA movement, the projected line of movement trajectory on x-y plane was parallel to the projection of strut which connects the moving node. Thus, the compressive force on the moving strut can be reduced after the FA movement. The two remaining candidates were then attached to the plane in sequence using the same principle. After all candidate nodes attached to the substrate and formed FAs, further extension of the spreading area were achieved by allowing the attached FAs to move on the x-y plane in radial orientation against the center of attachment area (Supplementary Fig. S1). The spreading principle of the COT structure was similar to the OT structure. The COT structure offered a maximum of 12 FAs. Therefore, two spreading types, with 8 and 12 FAs, were applied to examine the minimum energy stored in cytoskeletons during different stages of spreading. The spreading area was the area of the convex composed of the FAs. During each degree of spreading, at least three spreading examples were studied for both OT and COT structures.

To confine the tensegrity structure, several rules should be noted and complied during the simulation. Cables could not to stand compressive forces and carried zero force when the current length (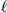) was shorter than the resting length (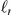). Unlike cables, struts were set for compression barring under regular conditions, but were still able to withstand tensile forces to prevent over-constrain and hardly-deformation. During deformation, free-constrained nodes should not sink into the x-y plane. When deformation violated the rules, new position(s) was sought for the assigned FA(s). Usually, only one FA was moved to the designated position at each FA movement. If assignment of only one node cannot find the suitable simulation outcome, several nodes with similar height were assigned simultaneously to new locations by following the aforementioned rules. When deformation violated the rules, new position(s) in radial direction was modified for the assigned FA(s).

### Calculation of Force and Strain Energy

The initial self-equilibrated tensegrity structure had several initial boundary conditions as initially attaching on the substrate. In current study, the initial boundary condition was determined based on the potential for creating a larger and non-uniform spreading morphology (Figs. 1A and 1C). The strain energy stored in each cable and strut can be calculated using their carried force (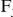 in Eq.(1) and 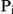 in Eq.(2)) and axial deformation (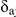 and 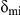).

The total energy of the cytoskeleton, U, then become:
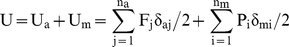
(3)where 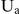 denotes the energy stored in cables, and 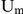 denotes the energy stored in the struts. 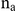 and 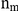 denote the numbers of cables and struts in a tensegrity structure, respectively. The OT structure has 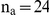 and 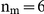 while the COT structure has 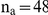 and 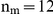. The COT structure was more complex and had two types of spreading. Thus, a polynomial was applied to fit the optimized energy curves among the selected simulation results. The r-Square was the criterion to determine the order of the polynomial curves.

## Results

### Tensegrity Structures with Different Complexities

Two different complexities of tensegrity structures, OT (Figs. 1A–B) and COT (Figs. 1C–D), were established in a round shape with various numbers of cables (red, Figs. 1A, 1C) and struts (blue, Figs. 1A, 1C). To simulate realistic conditions, the original heights (H_0_) of both tensegrity structures were approximately 14∼15 µm (Figs. 1A, 1C) in accordance with the diameter of human cells *in vitro* [27]. The material properties of actin filaments and microtubules were assigned using *in vitro* experimental results (Table 1) [23]. The numbers of candidate nodes were 6 and 12 in the OT and COT structures, respectively (red circle, Figs. 1B, 1D). When the attached nodes reached the substrate, the focal adhesions (FAs) formed to transmit intracellular forces to external substrate.

### Octahedron Tensegrity Unable to Spread Out

**Figure 2:**
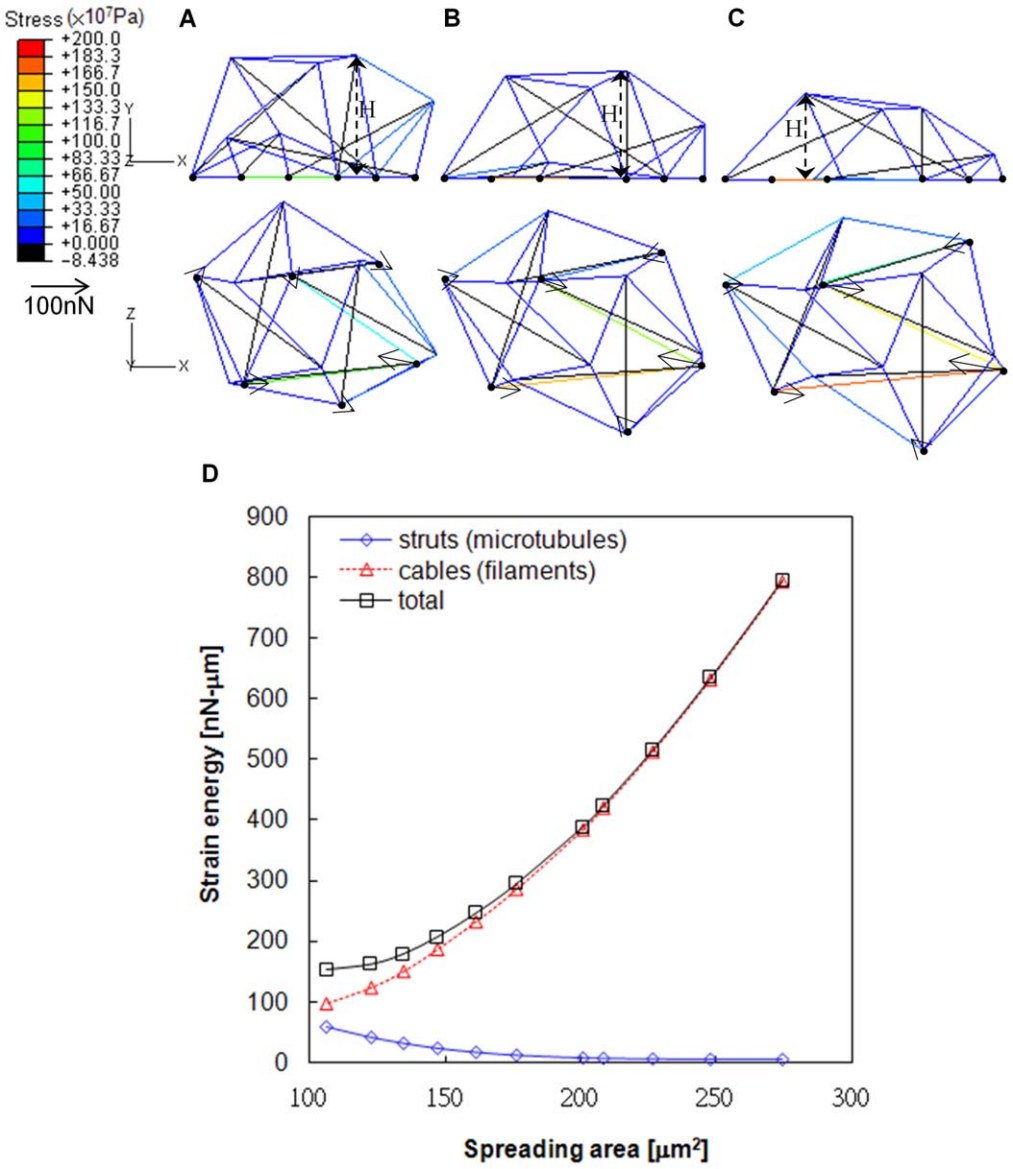
Decline of stored energy in struts limited the spreading of the OT structure. Spreading morphology, arrangement of cytoskeleton, and distribution of traction force are shown during different spreading stages of the OT structure with a remaining height of 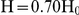 and a spreading area of 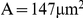 (A), 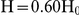 and 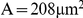 (B), and a lowest height of 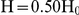 and maximum spreading area of 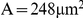 (C). The color bar denotes the magnitude of stress carried in cables indicating the tension in actin filaments. The arrowhead direction represents the direction of traction force and the length denotes the magnitude of force on focal adhesions (FAs) (A–C). The total energy of the cytoskeleton (squares) was contributed mainly by cables, especially where the spreading was significant (D). The increase of strain energy stored in cables (triangles) indicates that the tension rose in actin filaments as the OT structure spread out, while the stored energy in struts (diamonds) declined to zero and limited the spreading by instable microtubules with bearing no compression (D).

In the OT structure, the original attachment comprised three nodes. The additional three FAs were immediately attached to the rigid floor and reached the maximum spreading areas. Three different degrees of cell spreading for intermediate configurations of the same simulation (Figs. 2A–C) indicated the cell height (top) and the spreading area (bottom) after deformation. The color bar denoted the value of stress carried in the constitutive elements for both cables and struts. Arrowheads represented the direction of traction forces and the length of arrows demonstrated the magnitude (Figs. 2A–C, bottom). The traction force increase positively correlated with cell spreading. The strained energy in actin filaments (cables) increased as the cells spread out, whereas the strain energy in microtubules (struts) declined to zero and limited cell spreading (Fig. 2D). The spreading simulation of the OT structure was restricted at the extreme spreading area (274µm^2^, supplementary Fig. S2) that was still much smaller than the spreading area in living cells [27], [28]. The descending curve for the strain energy in struts demonstrated no reverse opportunity and became subject to tension as the structure was forced to further spreading (Fig. 2D). Thus, the instability of struts was the main reason to limit cell spreading in the OT structure.

### Cuboctahedron Tensegrity Represents Cell Spreading

**Figure 3:**
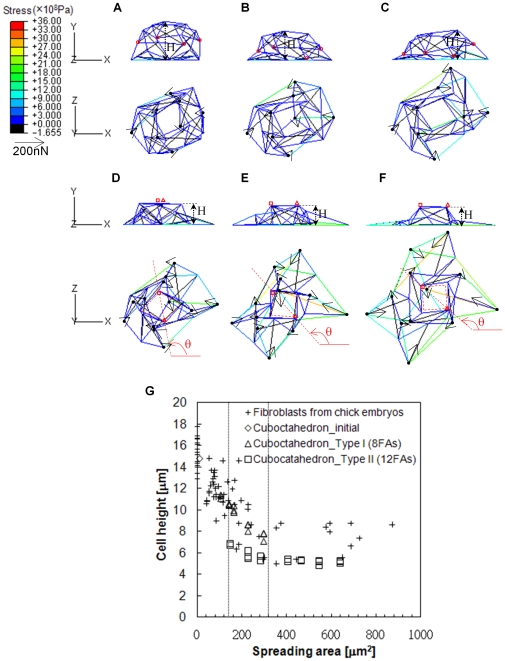
COT structure represents virtual cell morphology and force distribution with different degrees of spreading. Type I spreading, using 8 FAs, was demonstrated in three degrees of spreading states with 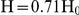 and 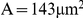 (A), 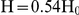 and 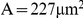 (B), and 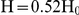 and 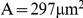 (C). Type II spreading used 12 FAs and the spreading area significantly increased with 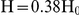 and 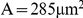 (D), 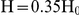 and 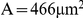 (E), and 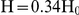 and 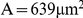 (F). The traction forces on FAs increased in both spreading types. Similar to living cells, the centripetal direction of traction forces occurred at peripheral FAs and the outward direction of traction forces occurred at inner FAs in the spread out COT structure (F). The partial rotation of the uppermost layer (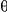) in type II spreading reduced the intracellular tension and enlarged the spreading area (D–F). The simulated cell height against the spreading correlated with the experimental data from chick fibroblasts [27] (G).

The COT structure was adapted for cell spreading by comprising twice the amount of cytoskeletal elements and nodes than the OT structure. The maximum 12 FAs in the COT structure divided the spreading status into two conditions, type I 8 FAs (Figs. 3A–C) and type II with 12 FAs (Figs. 3D–F). The dynamic processes of cell spreading were demonstrated in the COT structure with type I (supplementary Movie S1) and type II (supplementary Movie S2) spreading conditions. The COT structures spread in random radius directions and demonstrated the spreading cases of 45% (Figs. 3A, 3D), 75% (Figs. 3B, 3E), and 100% (Figs. 3C, 3F) of the spreading area. The complex COT structure contained three layers and partial rotation of the uppermost layer structure reduced the intracellular stress for further enlargement of spreading areas. The diagonal line of the uppermost trapezoid and the x-axis carried the rotation angle (θ) during spreading (Figs. 3D–F). The height of the COT structure decreased when the attaching area spread out. The numbers of cables on the substrate surface increased and carried greater tensile force than upper cables in the spread out COT structure (Figs. 3A–F). The height and attachment area in the COT structure significantly correlated with experimental results in fibroblasts [27] (Fig. 3G). The simulated results were consistent with *in vitro* observations that thin actin is distributed on the cortex of cells, while strong stress fibers are arranged on the base of cells [29]. Furthermore, the traction force on FAs provided a more delaminate distribution in the COT structure. The forces were usually larger and oriented toward the centripetal on the peripheral FAs, but pointed outward with less traction force on the inner FAs (Fig. 3).

**Figure 4:**
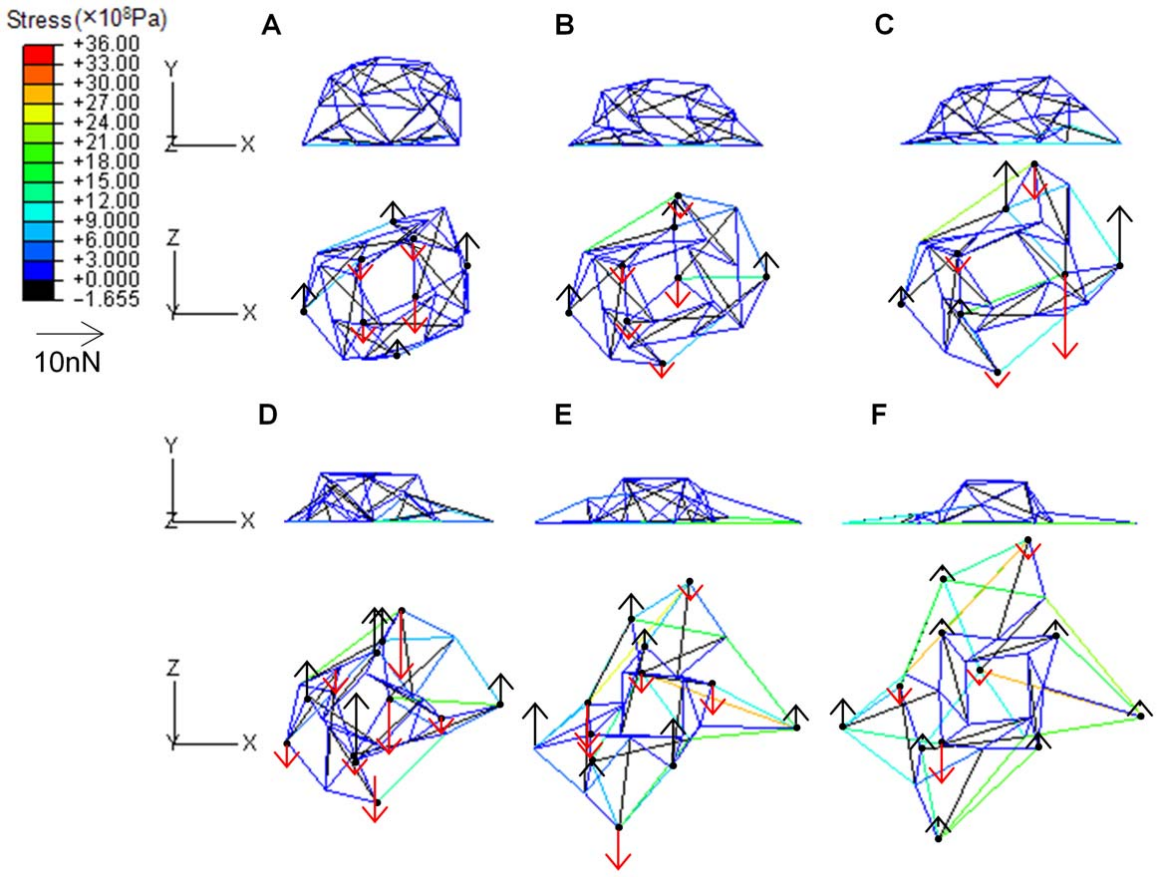
COT structure simulating normal force in Z direction. The arrow-head demonstrates the direction (also represented by black and red color for pulling and compressing force on substrate, respectively) and the length indicates the magnitude of normal forces exerted on the substrate through FAs. The pulling force at peripheral FAs and the compressive force in the central region of the spread out COT structure demonstrate 3D force interactions with the substrate (F).

The forces in tangential (XY) and normal (Z) directions were further analyzed in the COT structure (Fig. 4). The FAs not only exerted the tangential force within cell (traction force as aforementioned), but also the normal force applied to the rigid floor. In small spreading areas, normal forces were upward at the cell edge and downward in the central region (Fig. 4A). Subsequently, the distribution of normal forces varied with the degree of spreading. The magnitudes of normal forces, as indicated by arrow length, were much smaller than the tangential forces. The declined of normal force was more obvious when larger spreading area occurred with 12 FAs in the COT structure (Figs. 4D–F).

**Figure 5:**
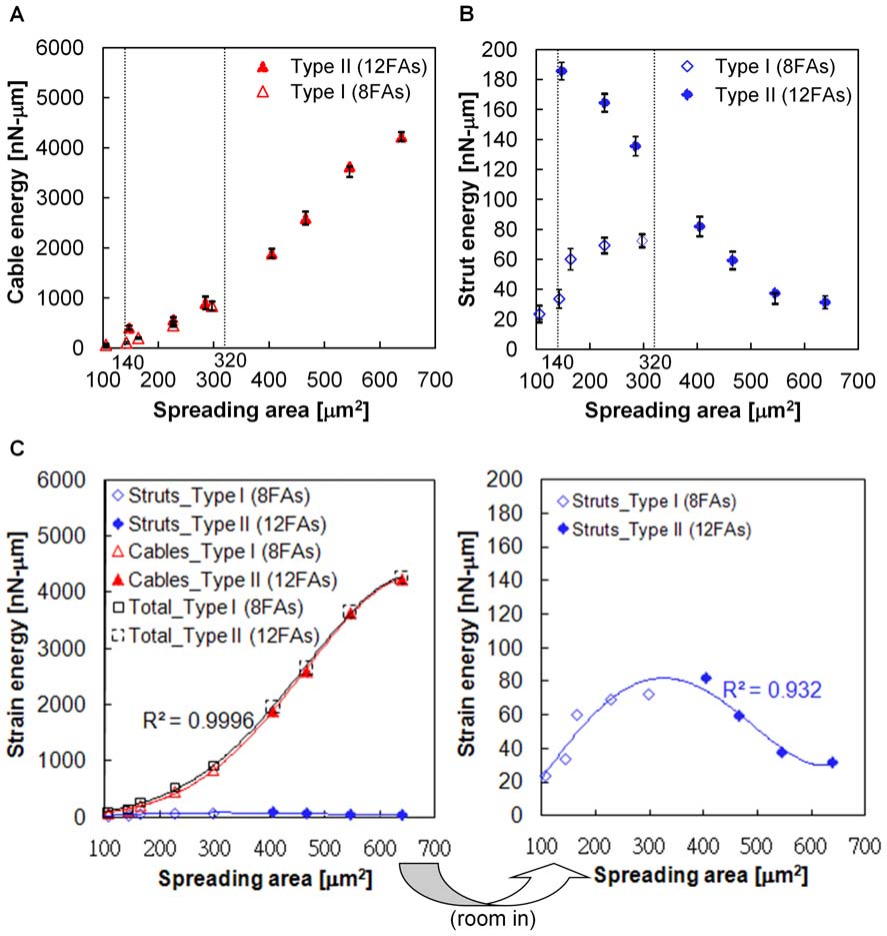
The dynamics of strain energy in the cytoskeleton during spreading of the COT structure. The energy stored in cables increased with the enlargement of the spreading area for both spreading types (A). The stored energy in struts increased nonlinearly in type I spreading (open diamonds) (B). Using type II spreading with 12 FAs (solid diamonds) caused higher strut energy than type I in the beginning of spreading, but declined as the cells spread out. The energy of cables (A) and struts (B) is presented by mean ± standard deviation. An overlapping region (between the spreading area of 140–320µm^2^) suggests the optimized energy should be considered by minimizing energy consumption. Optimized energies were estimated by fitting lower energies with the four-order polynomial curves (blue for strut, red for cable, and black for whole structure) (C). The right figure illustrates enlargement of optimized strain energy in struts.

The energies stored in the cytoskeleton and their constitutive elements were calculated according to the forces and deformations supported in cables (Fig. 5A) and struts (Fig. 5B). The energy curves of cables and struts overlapped between two types of spreading deformations (between the dash vertical lines in Figs.5A and 5B) and indicated an optimized result for different FA numbers in the spreading simulation of the COT structure. By selecting the lower-energy data points in the overlapping region, the optimized energy curves in cables and struts were obtained by fourth order multi-point fitting for different degrees of cell spreading (Fig. 5C). The r-Square for the fitting curves of cables, struts, and total energy were 0.9996, 0.932, and 0.9996, respectively. The COT structure solved energy decline problems in struts during OT simulations. The energy in struts increased after initiation and then decreased against the increase of the spreading area (Fig. 5B). The fitting curves tended to have stable states in the end. The total energy increased nonlinearly and dominantly contributed by cables in the simulated structures.

### Independence of mechanical parameters for octahedron spreading

**Figure 6:**
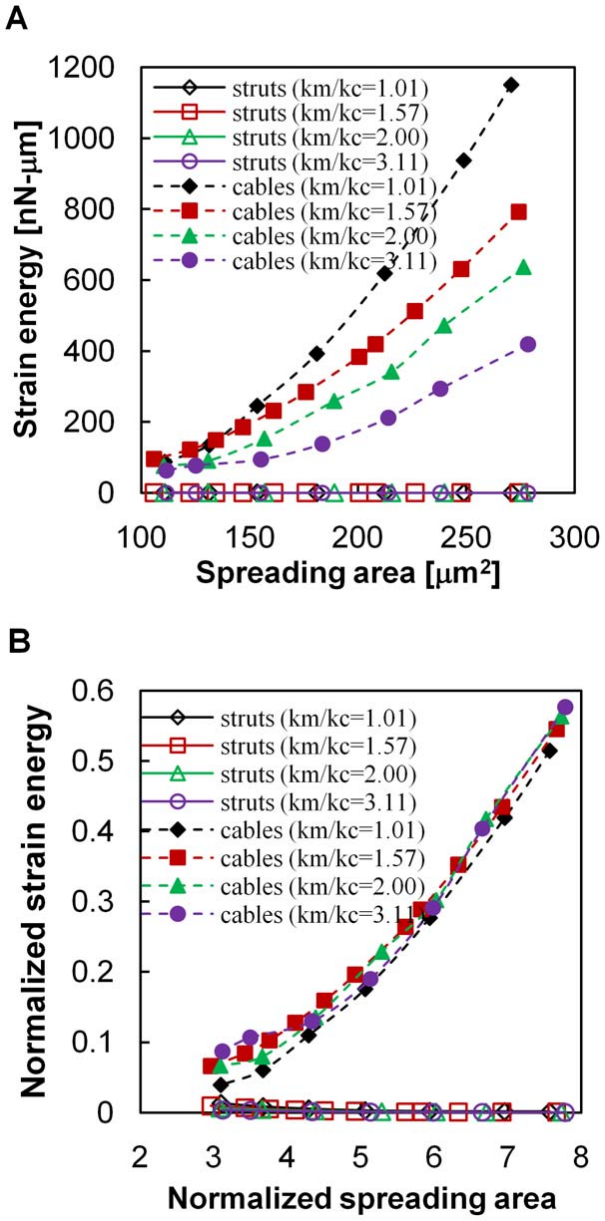
Changing the relative material properties among cables and struts does not enlarge spreading in the OT structure. The energy stored in both struts (empty marks) and cables (solid marks) decreased and indicated easier structure deformation when the ratio of axial stiffness between struts (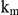) and cables (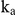) increased (A). Changing the 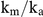 ratio did not restore the abolishment of strut energy and still limited the spreading of the OT structure. Normalization of Young's modulus, element dimensions, and the spreading area demonstrate the effects of changes in the 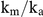 ratio better at the beginning of spreading (B).

In the OT and COT structures, cables and struts were constituted in according with measured material properties of actin filaments and microtubules. However, values of material properties vary in different cells and/or measure methods [23], [30]. To ascertain the effect of material properties on cell spreading, four different axial stiffness ratios of struts (k_m_) and cables (k_a_), k_m_/k_a_ = 1.01, 1.57, 2, and 3.11, were adopted in the numerical analyses of the OT structure (Fig. 6). A larger k_m_/k_a_ indicates easier deformation in cables than in struts. The energy stored in both cables (filled markers) and struts (empty markers) decreased with an increasing k_m_/k_a_ ratio (Fig. 6A). The changing k_m_/k_a_ ratio did not solve the limitation of energy abolishment in struts during spreading indicating that energy distribution between cables and struts is independent of the k_m_/k_a_ ratio in the OT structure.

Young's moduli of cables (E_a_) and struts (E_m_) also covered a wide range of documented values [23], [30], [31], [32]. The energy stored in cables and struts was further normalized by their own Young's modulus and dimension (E_a_A_a_l_0_ and E_m_A_m_L_0_). The spreading area was also normalized by the initial attaching area. The normalization separated the effect of various k_m_/k_a_ ratios at the initial spreading stages, but diminished as the cell spread out (Fig. 6B). These results implied that cell could deform easier as store less energy by changing the material property of cytoskeleton, especially k_m_/k_a_ ratio. However, the OT structure was still insufficient to describe cell spreading due to the abolishment of compressive energy in struts.

## Discussion

The dynamics of cytoskeletal spreading and energy arrangement in both actin filament and microtubule were demonstrated by simulating the tensegrity structures in present study. Among various stimulatory models related to actin filaments, the elastic modulus of cell was estimated based on the bending of actin element in the open-cell foam model [12]. The 3-D prestressed cable net was used to indicate the distribution of stretching cables during cell migration [4]. A single constituent (actin filament) was considered to represent cell properties in these above models. The granular model could vividly mimic the force topology for well-spread cells by various granules, elastic springs, and rods to indicate interconnections, actin filaments, and microtubules, respectively [9]. However, mechanical properties used in the granular model did not yet correspond to those of living cells. The tensegrity model was verified to have several features consistent with living cells, such as cell stiffening or softening, high-traction force with microtubules disruption, and non-liner mechanical responses [1], [2], [7], [15], [17], [20], [21], [26], [33], [34]. The tensegrity concept may also simulate the cell nucleus and stress fibers [2], [3], [35], [36]. Verifying the simulations using *in vitro* experiments could improve the precision of analytical results, such as the variation in cell stiffness against the degree of cell spreading [19]. The credible spreading structures found in the COT structure could investigate spreading-associated mechanical behaviors of cells. In dynamic spreading, the COT structure provided superior results to the OT structure by comparing the spreading area, stored energy and force distribution. The multiple-layer COT structure contributed a larger spreading area by partial rotation of the uppermost layer (Figs. 3D–F). The maximum spreading area without rotation of the uppermost layer was much smaller (340µm^2^, Supplementary Fig. S3) than rotation of 61 degrees on the uppermost layer (639µm^2^, Fig. 3F). Rotation of the uppermost layer rearranged the force distribution between cable and struts that reduced the instability of struts to provide further spreading. However, whether living cells also reduce intracellular instability by rotation is still unknown. The height of COT structure can also be influenced directly by the uppermost layer; the height decreases when the FAs reach outward. The simple OT structure could not simulate layer rotation, because all struts attached one end on the substrate right after the initiation of spreading. The detailed force profiles of struts in the OT structure were further assessed during spreading (Supplementary Fig. S4). The forces carried in all six struts decreased and confirmed the structural instability of the OT structure. Together with the outcome in changing the k_m_/k_a_ ratio (Fig. 6), the restriction of struts was a main factor for limiting spreading of the OT structure. The COT structure is the most complex tensegrity structure that can form sphere-like cell morphology in the initial state. When the structure complexity further increased, the tensegrity structure was more similar to a cylinder, which is not cell geometry in suspension [18].

Traction forces were exerted by the deformation and rearrangement of cytoskeleton on the substrate via FAs. The direction of traction forces in the COT structure were consistent with observations in living cells [28], [37]. Moreover, doubling the FA numbers in the COT structure generated traction forces in the central region of the spreading area. The traction forces rose with increasing deformation in living cells [38]. In the current study, increasing the tensile forces carried in cables resulted in the increase of traction forces. The traction forces exerted on the attaching substrate through the FAs are 3-D in living cells, because the cell is a 3-D structure [39]. The traction forces in tangential and normal directions were measured using a polyacrylamide deformable substrate in bovine aortic endothelial cells. Although the substrate was assumed to be a rigid plane in the present study, the normal force in the simulated COT structure with a small spreading area may be similar to the experimental results found upward at the cell edge and downward under the nucleus (Fig. 4A) [39]. The partial inconsistency with the increasing spreading area (Fig. 4) may be influenced by the rearrangement of structure and the effect of nucleus in living cells. The energy curves in the OT and COT structures indicated that the tensile actin filaments contribute to the major strength of cells, while the compressive microtubules stabilize the cell structure (Figs. 2D and 5). The struts consumed only 0.1–6.5% (average was 3%) of the total stored energy, but were important for stabilization of the intracellular structure during COT deformation. The role of microtubule was supported by *in vitro* observations that microtubules balanced the tension (3∼13%) carried in actin filaments [14]. As cells flattened and spread out, actin filaments carried larger tensile forces and could partly equilibrate themselves. The cytoskeleton was closer to a tension structure and the responsibility of microtubules was mitigated in a well-spreading COT structure. Previous studies also suggested other mechanical parameters, such as pre-tension in actin filaments [15], [20], volumetric density, and dimensions of cytoskeletal constituents [25], buckling/rupturing property of microtubules [1], [7], [40], and bending property of actin filaments [12]. Here, different “initial” pre-forces of F_0,tri_ = 40Pn, F_0,squ_ = 45.7Pn and P_0_ = 96.1pN were applied to evaluate the effect on simulated results in the COT structure. Undergoing similar designated spreading, different pre-forces resulted in tiny differences (within 1%) in intracellular force and stored energy (Supplementary Fig. S5). The effect of tension in actin filaments was reflected by the degree of spreading in current study. However, all elements in tensegrity structure must be validated based on the hierarchical system in a tensegrity structure [2], [35], [36] where each cable (actin filament) or strut (microtubule) can be represented by another tensegrity structure composed of shorter cables and struts (Fig. 8 in [2]). Therefore, some mechanical parameters, such as microtubule buckling and rupturing, playing a decisive role in force equilibrium and rearrangement of cytoskeleton could be temporarily neglected in the current study. The effects of buckling and rupturing microtubules on cell mechanical responses were investigated in previous studies with a tensegrity structure [1], [7], [41]. In future study, consideration of more mechanical parameters in different structure hierarchical system into the spreading cytoskeleton models developed in the study is suggested for mimic all the possibility of cell response. In the current study, the lower axial stiffness of actin filaments allowed the simulation to reach an expected spreading area with carrying less force; whereas higher axial stiffness in microtubules supported the structure with less axial deformation and reduced the strain energy of the cytoskeleton (Fig. 6A). This further emphasized the role of microtubules in structure reorganization and stabilization.

Two types of spreading in the COT structure provided superior results among eight and twelve FAs in different stages of spreading (Fig. 5C). However, more spreading types with different FA numbers may provide more details and more precise energy curves. Stable-guaranteed spreading according to natural folding of structure deformation was used to choose two spreading types in the COT structure. Insufficient elements number to mimic the vivid cell cytoskeleton network might occur in the COT structure. Therefore, future studies may verify the COT simulations using *in vitro* labeling of cytoskeletons in living cells. Other limitations might occur using the COT structure to simulate cell behaviors. Current tensegrity structures are macroscopic and do not incorporate effects of dynamic fluctuations of the cytoskeleton, such as assembling and dissembling. However, COT simulations can reveal the force distribution in a cell (as a macroscopic unit) and then predict possible remodeling dynamics in specific parts of the cell. Understanding structure interactions within whole cell is important for intracellular force dynamics to capture cell features and investigate related molecular mechanisms. The COT structure can simulate dynamic cell behavior and thus provides an important tool to improve research of structure interactions within whole cells.

## Supporting Information

Figure S1 The radial orientation against the center of attachment area for a FA movement.
(0.97 MB TIF)

Figure S2 Spreading morphology and traction distribution of an extreme spreading in the OT structure. The spreading area of 274µm^2^ is much smaller than in documented cell data.
(2.58 MB TIF)

Figure S3 Twice the number of FAs is insufficient to contribute to cell spreading without uppermost layer rotation in the COT structure. The maximum spreading area with 12 FAs reached only 340µm^2^ without layer rotation (A). The maximum spreading of the COT structure almost doubled, when the rotation of the uppermost layer was simulated with an angle (θ) (B).
(3.14 MB TIF)

Figure S4 The forces carried in all six struts decreased while the OT structure spread out. Many struts bore zero force and limited the structure from further spreading.
(0.72 MB TIF)

Figure S5 The comparison of strain energy and traction force between two different initial pre-force conditions. The spreading area of 227µm^2^ (A–B) and 545µm^2^ (C–D) was simulated using 12 FAs in the COT structure, but different initial pre-tensions (F_0,tri_). The strain energy (A and C) and traction force (B and D) did not significantly differ among different pre-force conditions.
(0.89 MB TIF)

Movie S1 Dynamic simulation in type I spreading of the COT structure. The final outcome is shown in Fig. 3C.
(3.99 MB MOV)

Movie S2 Dynamic simulation of the COT structure with type II spreading. The final outcome is shown in Fig. 3F.
(4.59 MB MOV)
