# Supplementary figures and images for: Complexity of the Tensegrity Structure for Dynamic Energy and Force Distribution of Cytoskeleton during Cell Spreading
Source: PLoS One. 2010 Dec 21;5(12):e14392. doi: 10.1371/journal.pone.0014392 (PMC3006198; doi:10.1371/journal.pone.0014392)

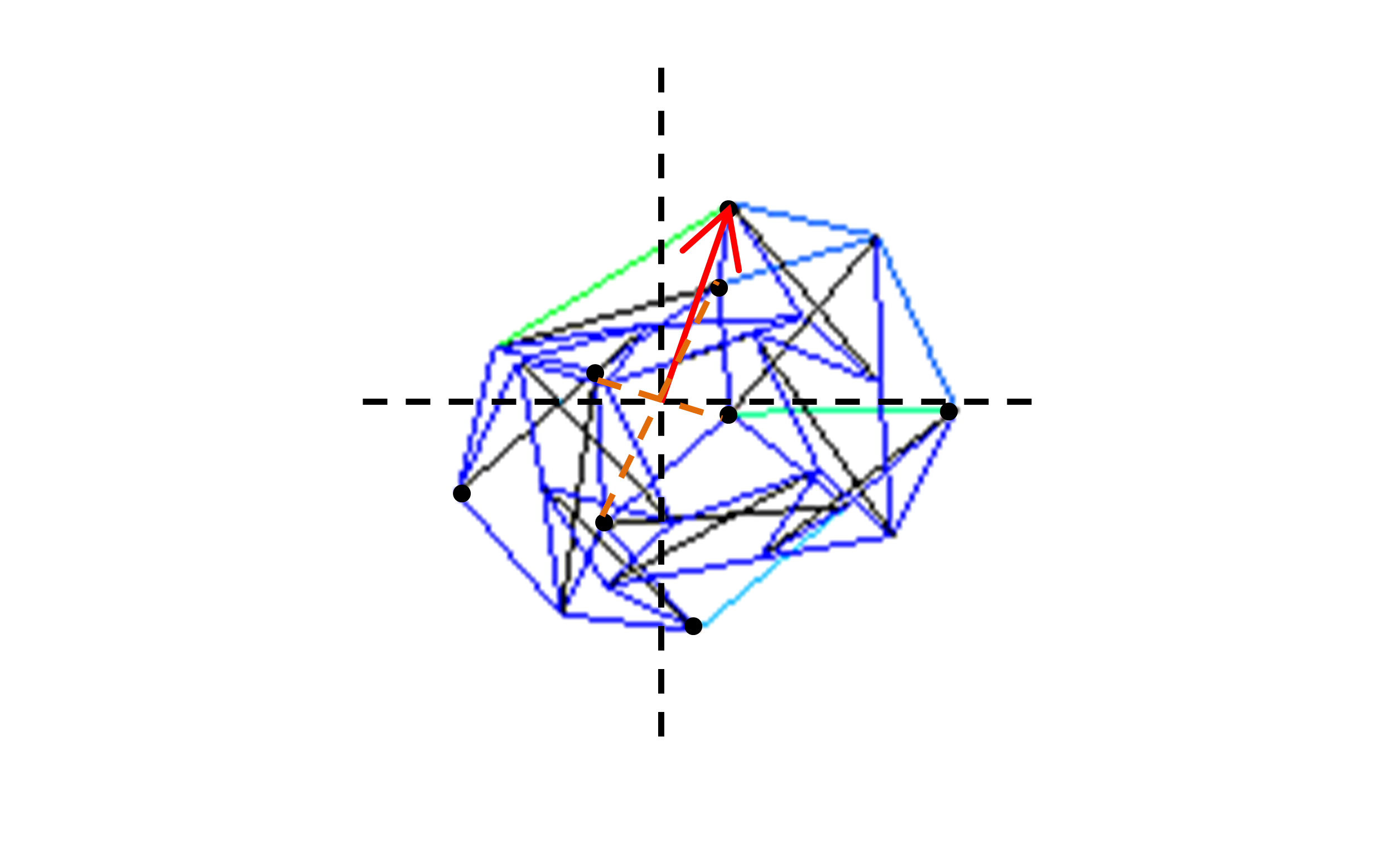

Supplement: Figure S1 — The radial orientation against the center of attachment area for a FA movement. (0.97 MB TIF) [file pone.0014392.s001.tif]

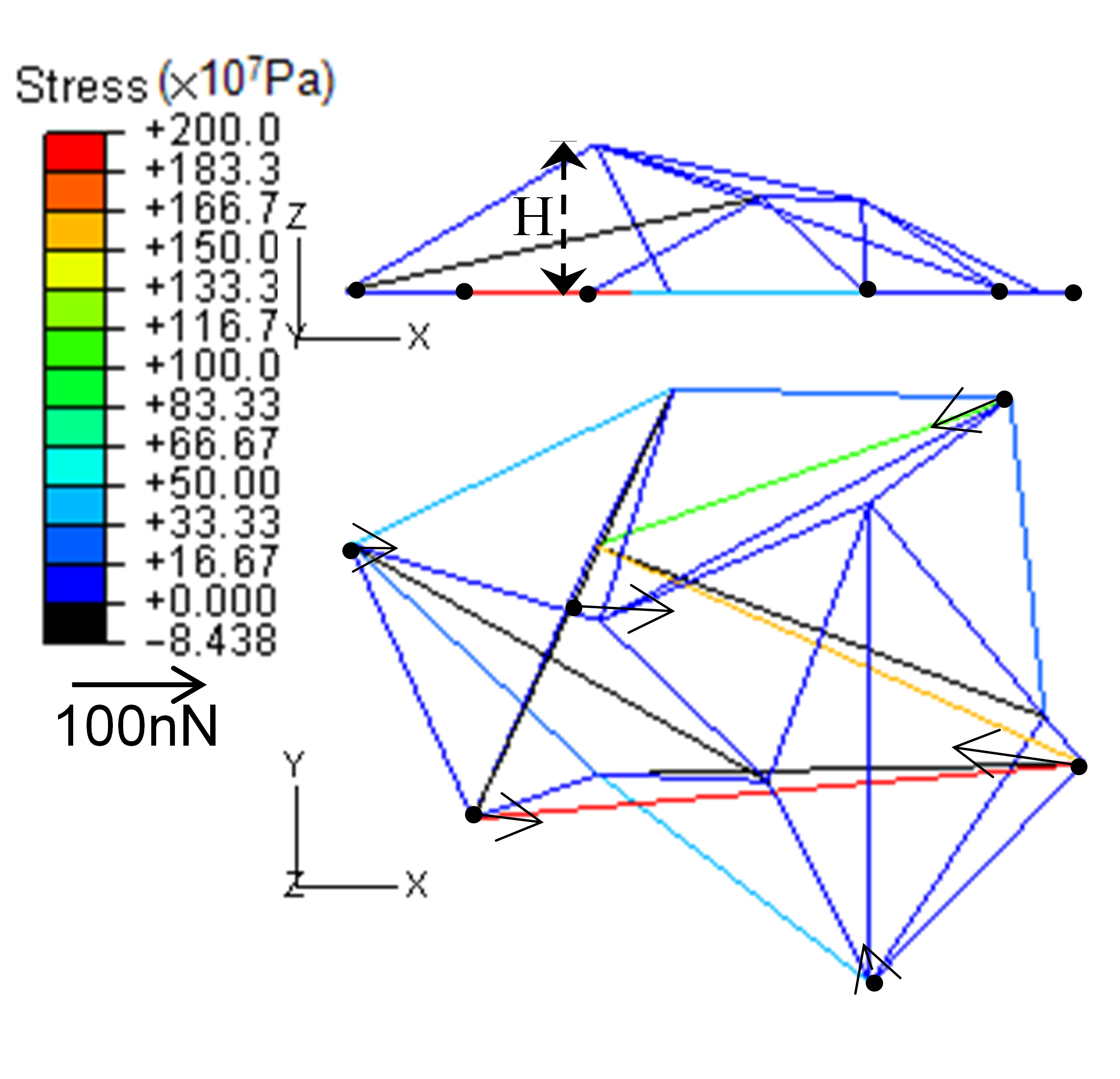

Supplement: Figure S2 — Spreading morphology and traction distribution of an extreme spreading in the OT structure. The spreading area of 274µm2 is much smaller than in documented cell data. (2.58 MB TIF) [file pone.0014392.s002.tif]

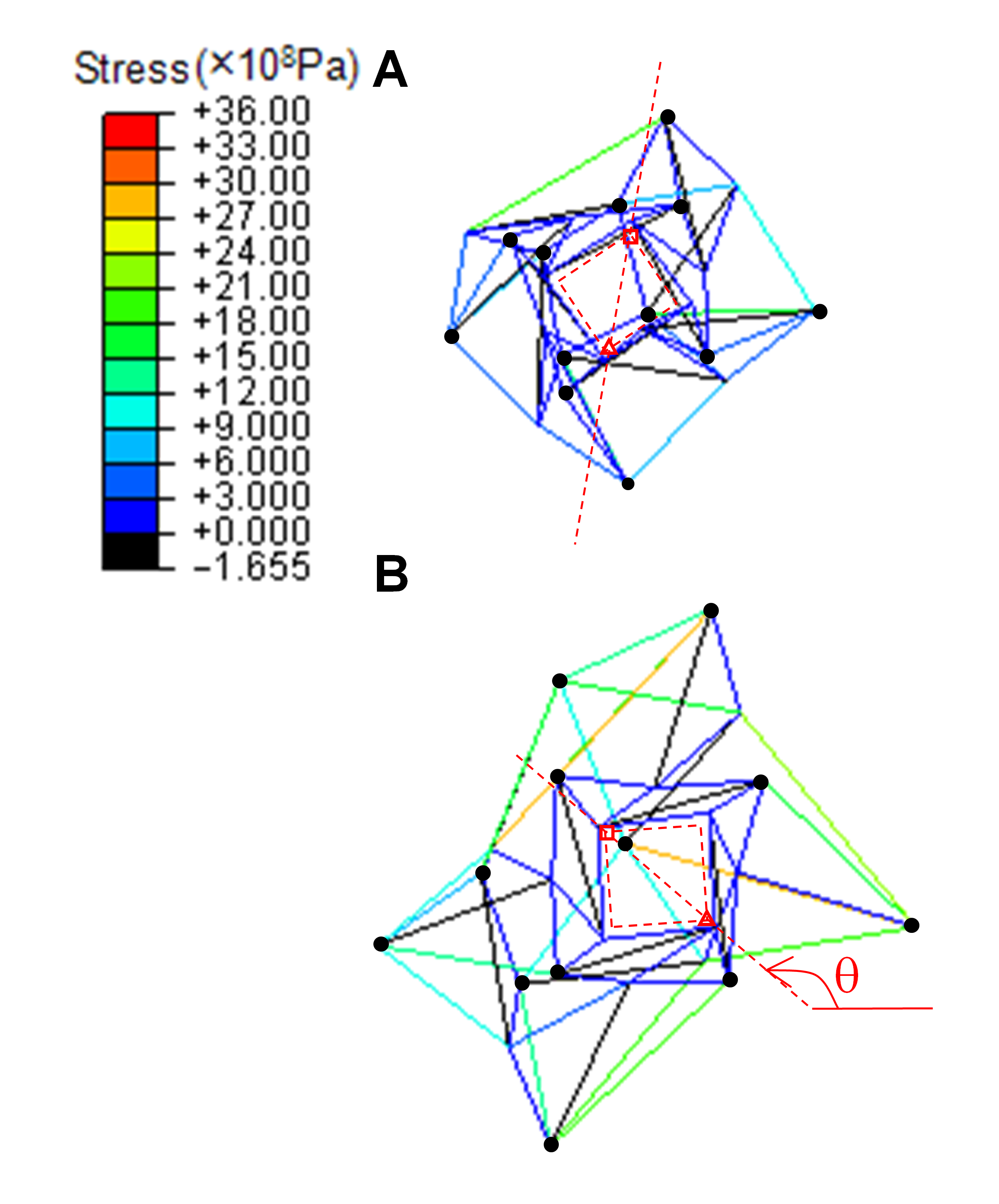

Supplement: Figure S3 — Twice the number of FAs is insufficient to contribute to cell spreading without uppermost layer rotation in the COT structure. The maximum spreading area with 12 FAs reached only 340µm2 without layer rotation (A). The maximum spreading of the COT structure almost doubled, when the rotation of the uppermost layer was simulated with an angle (θ) (B). (3.14 MB TIF) [file pone.0014392.s003.tif]

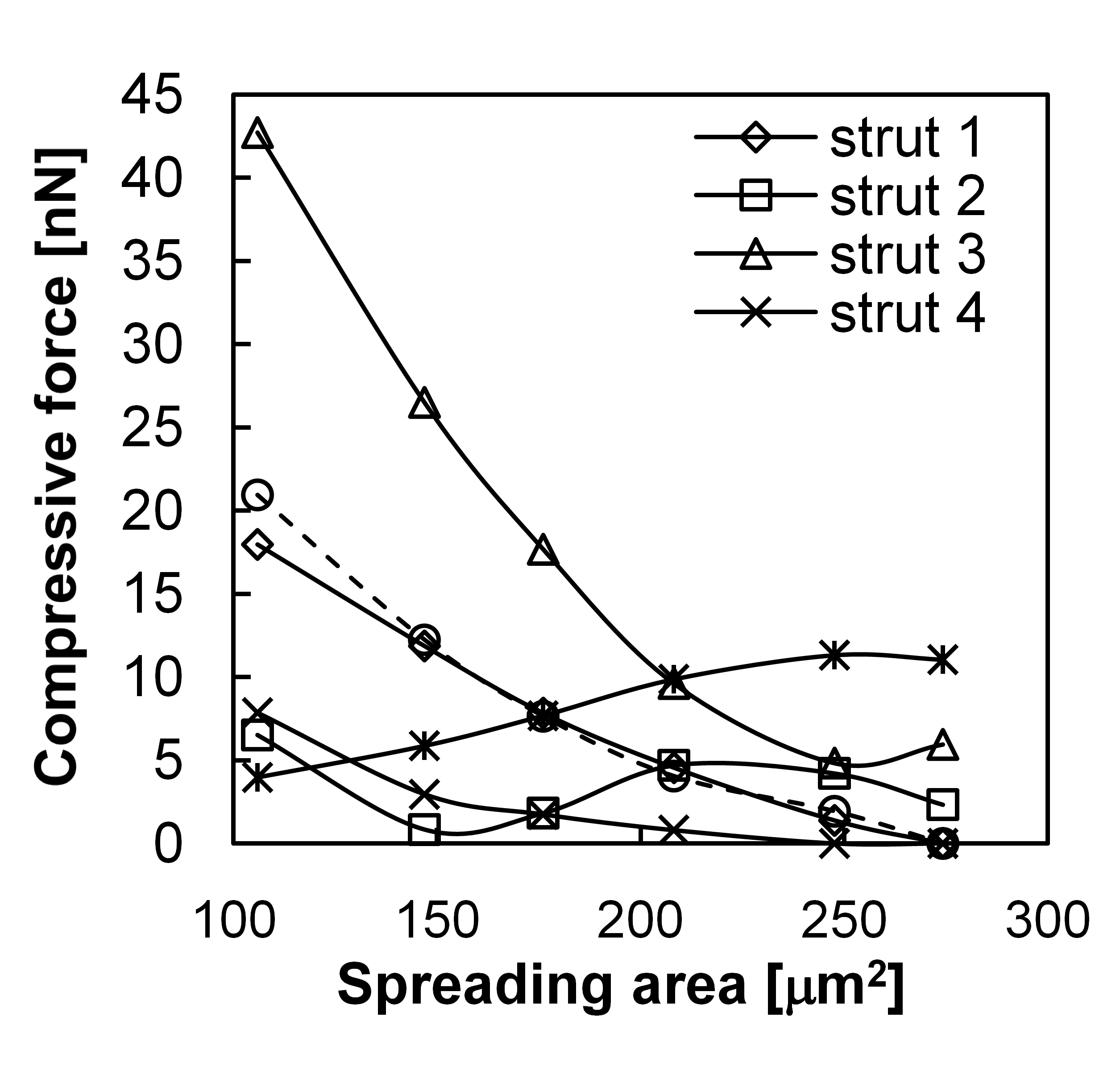

Supplement: Figure S4 — The forces carried in all six struts decreased while the OT structure spread out. Many struts bore zero force and limited the structure from further spreading. (0.72 MB TIF) [file pone.0014392.s004.tif]

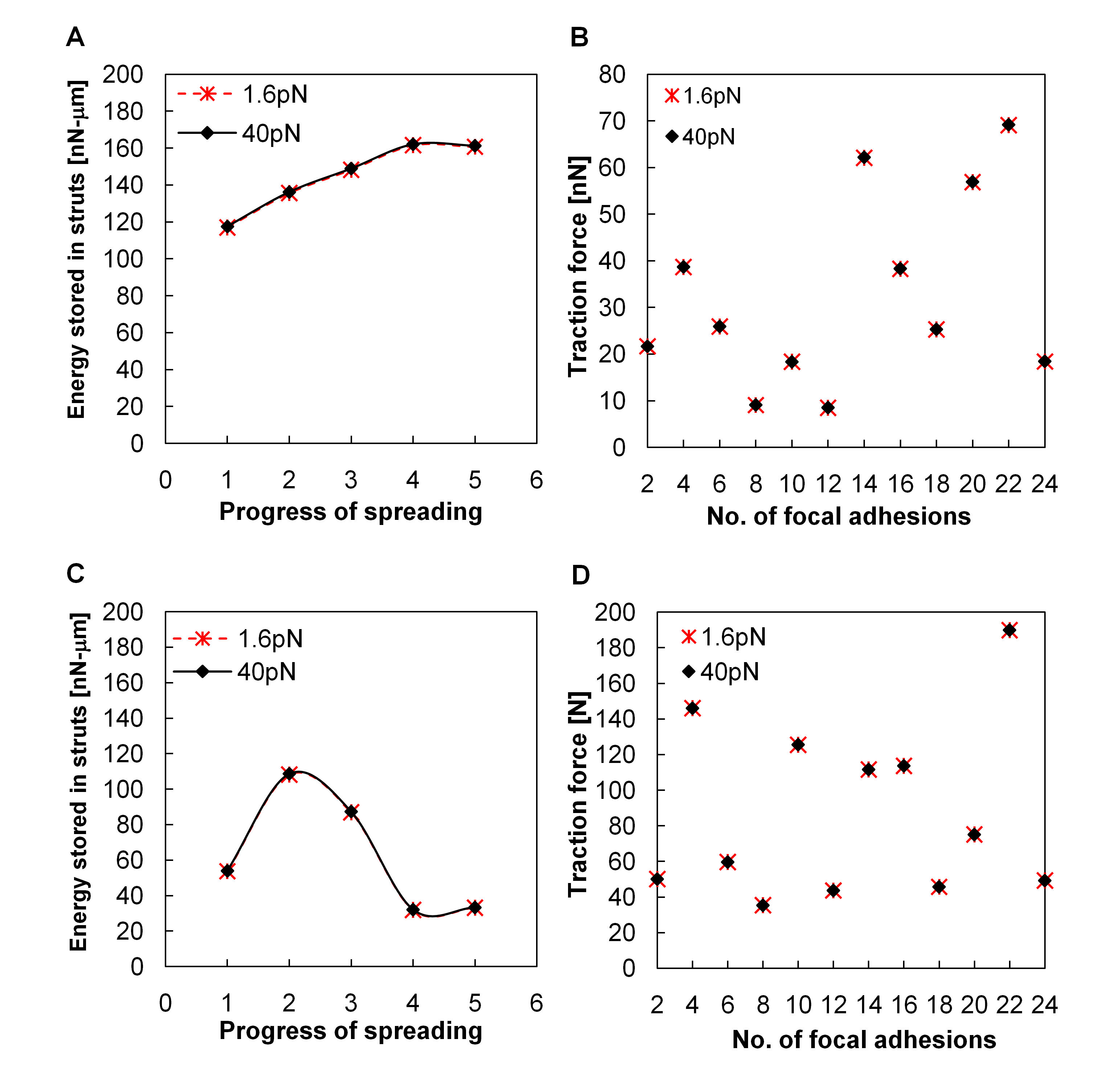

Supplement: Figure S5 — The comparison of strain energy and traction force between two different initial pre-force conditions. The spreading area of 227µm2 (A–B) and 545µm2 (C–D) was simulated using 12 FAs in the COT structure, but different initial pre-tensions (F0,tri). The strain energy (A and C) and traction force (B and D) did not significantly differ among different pre-force conditions. (0.89 MB TIF) [file pone.0014392.s005.tif]
